# Analysis of *SIRT1* genetic variants in young Mexican individuals: relationships with overweight and obesity

**DOI:** 10.3389/fgene.2024.1278201

**Published:** 2024-04-04

**Authors:** S. Salazar-García, A. Ibáñez-Salazar, E. Lares-Villaseñor, Noemi Gaytan Pacheco, E. Uresti-Rivera, D. P. Portales-Pérez, U. De la Cruz-Mosso, J. M. Vargas-Morales

**Affiliations:** ^1^ Laboratorio de Análisis Clínicos, Facultad de Ciencias Químicas, Universidad Autónoma de San Luis Potosí, San Luis Potosí, México; ^2^ Unidad Académica de Ciencias Químicas, Universidad Autónoma de Zacatecas ¨Francisco García Salinas, Zacatecas, México; ^3^ Laboratorio de Biología, Facultad de Ciencias Químicas, Universidad Autónoma de San Luis Potosí, San Luis Potosí, México; ^4^ Centro de Investigación en Ciencias de la Salud y Biomedicina, Universidad Autónoma de San Luis Potosí, San Luis Potosí, México; ^5^ Red de Inmunonutrición y Genómica Nutricional en las Enfermedades Autoinmunes, Instituto de Neurociencias Traslacionales, Departamento de Neurociencias, Centro Universitario de Ciencias de la Salud, Universidad de Guadalajara, Guadalajara, México

**Keywords:** SIRT1, obesity, single nucleotide variants, genetic association, BMI, body mass index

## Abstract

The high prevalence of obesity in Mexico starting from the early stages of life is concerning and represents a major public health problem. Genetic association studies have reported that single nucleotide variants (SNVs) in *SIRT1*, an NAD^+-^dependent deacetylase that plays an important role in the regulation of metabolic cellular functions, are associated with multiple metabolic disorders and the risk of obesity. In the present study, we analyzed the effect that the SNVs rs1467568 and rs7895833 of the *SIRT1* gene may have on cardiometabolic risk factors in a young adult population from Mexico. A cross-sectional study was carried out with young adults between the ages of 18 and 25 who had a body mass index (BMI) greater than 18.5 kg/m^2^. This study included 1122 young adults who were classified into the normal weight (*n* = 731), overweight group (*n* = 277), and obesity group (*n* = 114) according to BMI of whom 405 and 404 volunteers were genotyped for rs1467568 and rs7895833 respectively using TaqMan probes through allelic discrimination assays. We found that the male sex carrying the G allele of rs7895833 had slightly lower BMI levels (*p* = 0.009). Furthermore, subjects carrying rs1467568 (G allele) showed a 34% lower probability of presenting with hyperbetalipoproteinemia where female carrying rs1467568 had lower levels of total cholesterol (*p* = 0.030), triglycerides (*p* = 0.026) and LDL cholesterol (*p* = 0.013). In conclusion, these findings suggest that the presence of both SNVs could have a non-risk effect against dyslipidemia in the Mexican population.

## Introduction

Obesity is a worldwide public health problem, and its prevalence is of particular concern in developed countries (Afshin et al., 2017; Secretaría de Salud Pública, 2018). The World Health Organization (WHO) defines it as an abnormal or excessive accumulation of fat that can be harmful to health (WHO, 2020). Obesity is a multifactorial disease associated with various cardiometabolic effects. For instance, an individual with a high body mass index (BMI) is at increased risk for type 2 diabetes (T2D), high blood pressure, cerebrovascular disease, sleep apnea, osteoarthritis, and certain cancers, such as endometrial, breast, and colon cancer (López-Gómez et al., 2016; Steele et al., 2017). Moreover, this disease may affect the patient’s health over a long duration, which can result in fertility problems, psychological problems and issues with self-esteem and failure in the later stages of development (Sánchez-Villegas et al., 2010; Cheng et al., 2016).

Sirtuins are a family of nicotinamide adenine dinucleotide (NAD)-dependent histone deacetylases, and therefore, their function is intrinsically linked to cellular metabolism. Sirtuins are important sensors of energy status, in addition to protecting cells against metabolic stress and regulating the aging process, and are themselves regulated by dietary and environmental stress (Chang and Guarente, 2014). Within the sirtuin family, the role of the seven mammalian sirtuins (*SIRT1–7*) has been determined, and sirtuin 1 (*SIRT1*) stands out as one of the most studied to date (Chang and Guarente, 2014; Bonkowski and Sinclair, 2016). Sirtuin-1, which is encoded by the *SIRT1* gene*,* is located in the nucleus and cytoplasm and plays an important role in epigenetic regulation by deacetylating a variety of transcription factors (Li et al., 2007). Therefore, it has been associated with processes such as aging, apoptosis, and inflammation. Additionally, it participates as a regulator of several cellular functions, such as stress (Naqvi et al., 2010), fat and glucose metabolism (Rodgers et al., 2005), gluconeogenesis and glycolysis (Frescas et al., 2005) and insulin secretion (Moynihan et al., 2005).

Interestingly, it has been observed how SIRT1 can regulate the expression of other anti-aging genes such as Klotho, p66Shc (longevity protein) and Forkhead box proteins (FOXO1/FOXO3a) and how various adverse environmental effects such as unhealthy diets can inactivate the SIRT1 gene which in turn could play a key role in the development of metabolic diseases and premature aging in individuals (Martins, 2016; Martins, 2017).

Additionally and consistent with the above, several single nucleotide variants (SNVs) in the SIRT1 gene have been reported to be linked to multiple diseases, including inflammation, obesity, type 2 diabetes, blood pressure, dyslipidemia, cancer and coronary artery disease as risk or non-risk factors (Zillikens et al., 2009; Ramkaran et al., 2016).

For example, an association of the *SIRT1* SNVs rs3758391 T/C [10:67883584 (GRCh38)] and rs369274325G/A [10:67912600 (GRCh38)] has been demonstrated with an increased risk of bladder cancer (Shafieian et al., 2019).), rs1467568 [10:67915401 (GRCh38)] with non-diabetic type 1 cardiorenal syndrome (Hou et al., 2019) and rs7895833 [10:67863299 (GRCh38)] with pediatric obesity as well as dyslipidemia in the geriatric population (Casarotto et al., 2019). While controversial other studies have not found an association of this SNV with metabolic syndrome (De Oliveira Meneguette et al., 2016).

Because in Mexico there is a high rate of obesity, especially among young adults, for whom this disease can affect their health for a longer period of time (Sánchez-Villegas et al., 2010; Cheng et al., 2016), our objective was to evaluate two genetic variants of *SIRT1*, the rs1467568 and rs7895833, and determine their association with cardiometabolic risk factors in a young adult population from the northeastern region of Mexico.

## Materials and methods

### Study group

A cross-sectional study was carried out with 1,122 students to the Universidad Autónoma de San Luis Potosí, (UASLP) from the state of San Luis Potosí, Mexico between 18 and 25 years old. The main criterion of selection was that they had a body mass index (BMI) greater than 18.5 kg/m^2^ and exclusion criteria included pregnancy, substance abuse, and cardiovascular disease history, cancer, acquired immunodeficiency syndrome, renal or liver disease, and being on treatment with hypoglycemic agents, antihypertensives, statins, or others.

The protocol was approved by the Ethics Committee of the Facultad de Ciencias Químicas, UASLP which has a Favorable Opinion for its operation from the National Bioethics Commission. It currently has the following Registration CONBIOÉTICA24CEI00320190726, with the registration number CEID2017060A. The study was designed according to the Declaration of Helsinki and ethical treatment of human subjects. All participants signed the informed consent letter and agreed to participate in the study.

Anthropometric, clinical and biochemical measurements: Glucose, cholesterol, triglycerides, HDL cholesterol and LDL cholesterol.

The students underwent a medical examination at the University Health Center of UASLP consisting of anthropometric measurements (height and weight) while systolic and diastolic blood pressure was measured in the dominant arm of the volunteer when seated position using a Welch Allyn^®^ Baumanometer according to clinical guidelines (NOM-030-SSA2-1999). The biochemical variables were determined in the Laboratorio de Análisis Clínicos de la Facultad de Ciencias Químicas UASLP. Glucose, cholesterol, triglycerides, HDL cholesterol and LDL cholesterol were quantified from blood serum samples (overnight fast 12 h) by spectrophotometric methods Mindray BS 300 Auto Chemistry Analyzer (Mindray^®^, Shenzhen, China).

The following cardiometabolic risk critical cutoff values were considered for this study according to National Cholesterol Education Program (NCEP) guidelines Adult Treatment Panel III (ATP III) and Mexican guidelines: hyperglycemia ≥100 mg/dL (NOM, 2003); hypercholesterolemia ≥200 mg/dL; hypertriglyceridemia ≥150 mg/dL; hyperbetalipoproteinemia ≥100 mg/dL (NOM, 2003); hypoalphalipoproteinemia ≤40 mg/dL in male and ≤50 mg/dL in female (Fabian, 2023) and systolic pressure ≥130 mmHg and diastolic pressure ≥85 mmHg (Ávila, 2011).

The participants were classified according to World Health Organization (WHO) guidelines to BMI into the following groups: SP (study population, *n* = 1122), OG (overweight group, *n* = 277; BMI greater than or equal to 25–29.9 kg/m^2^) and OBG (obesity group, n = 114; BMI greater than or equal to 30 kg/m^2^). We considered as a control group clinically healthy subjects with normal weight (NW, *n* = 731; BMI greater than or equal to 18.5–24.9 kg/m^2^) **(** Weir et al., 2023)

### Genotyping of genetic variants

Genomic DNA from all participants was extracted using a Promega^®^ commercial kit (A1125) and adjusted to a concentration of 10 ng/μL on a Sinergy BioTek^®^ spectrophotometer (USA) with a purity greater than 1.8 in absorbance ratio at 260 nm and 280 nm (A260/A280). Finally 405 and 404 volunteers were genotyped for rs1467568 and rs7895833 respectively. SNV detection was performed using TaqMan^®^ SNV genotyping allelic discrimination assays which were specific for *SIRT1* rs7895833 (4351379 C__29163689_10) and rs1467568 (4351379 C___1340398_10) (Thermo Fisher Scientific ^®^, Massachusetts, United States). All PCRs were run in triplicate and a no-template control was used for each single nucleotide variant. The Bio-Rad^®^ CFX96 Touch (Singapore) real-time PCR detection system was used. Allelic and genotypic frequencies were compared with the Ensembl project database [https://www.ensembl.org/index.html]. The SNVs rs1467568 and rs7895833 were chosen based on their frequency above 10% in the Latino population. These frequencies were obtained from data repositories such as NCBI dbSNP (https://www.ncbi.nlm.nih.gov/snp/).

### Statistical analysis

Descriptive statistics were calculated to determine the SNV prevalence of the OG and OBG groups, as well as biochemical and clinical risk factors. The normality of the data was determined using the Kolmogorov‒Smirnov test. To determine the significance of the proportions of the different categories, the chi-square test was used, and the Kruskal‒Wallis test and Mann‒Whitney *U* test were used for comparisons between groups. Genotypic and allelic frequencies of SNVs of the *SIRT1* gene and Hardy-Weinberg equilibrium (Excoffier and Slatkin, 1995) were determined. The calculation of linkage disequilibrium (LD) and haplotypic frequencies was performed using the SHEsis^®^ program (Shanghai Jiao Tong University, Shanghai Shi, China) based on the Excoffier-Slatkin expectation-maximization algorithm for maximum likelihood estimates. For the analysis of association, odds ratios (OR) were calculated using contingency tables and the chi-square statistic. In addition, for the SIRT1 variants we calculated the sample size using the Kelsey´s formula [Dean A.G., Sullivan K.M., Soe M.M (https://www.openepi.com/SampleSize/SSCC.htm)] with a statistical power of 80%, a 95% confidence interval (CI), and a maximum estimated odds ratio of 2 for low-penetrance SNV, resulting in 134 subjects for study group for the less frequent SNV (rs1467568 *SIRT1*).

## Results

### Anthropometric, clinical, and biochemical characteristics

A total of 1,122 young adults (523 women and 599 men) were analyzed. Most anthropometric, clinical and biochemical parameters were within normal ranges. A prevalence of overweight and obesity of 24.5% and 10.3%, respectively in both sexes and it was found that systolic and diastolic blood pressure, glucose, Triglycerides and Cholesterol HDL were different between men and women (*p* < 0.01) (Table 1).

**TABLE 1:** General description of the study population.

Parameter	Study population (n = 1122)	Male (*n* = 599)	Female (*n* = 523)	*p*
Demographics				
Age (years)^a^	20 (19–22)	20 (19–20)	20 (19–20)	-
Anthropometric characteristics				
Body mass index (kg/m^2^)^a^	23.3 (19.2–32.5)	23.5 (21.2–26.5)	23.1 (20.9–26.3)	0.136
Normal weight ^b^	731 (65.2)	378 (63.1)	353 (67.5)	0.182
Overweight ^b^	277 (24.5)	152 (25.4)	125 (23.9)	
Obesity ^b^	114 (10.3)	69 (11.5)	45 (8.6)	
Clinical characteristics^c^				
Systolic blood pressure (mmHg)	110 (80–160)	110 (80–160)	100 (80–140)	<0.001^*^
Diastolic blood pressure (mmHg)	70 (40–100)	70 (50–100)	70 (40–90)	<0.001^*^
Biochemical characteristics^a^				
Glucose (mg/dL)	85 (75–99)	87 (83–92)	84 (80–89)	<0.001^*^
Cholesterol (mg/dL)	161 (122–215)	161 (144–180)	162 (145–182)	0.823
Triglycerides (mg/dL)	86 (43–212)	90 (66–130)	82 (62–111)	<0.001^*^
Cholesterol HDL (mg/dL)	51.2 (35.6–132)	49.9 (42.5–57.8)	52.6 (46.1–61.3)	<0.001^*^
Cholesterol LDL (mg/dL)	89.9 (55.6–132)	90.5 (75.1–106)	89.4 (76.8–105)	0.818

*SIRT, 1*: Sirtuin 1. The cutoff criteria were based on the World Health Organization where normal weight: body mass index ≥18.5 kg/m^2^; Overweight: body mass index ≥25 kg/m^2^; Obesity: body mass index ≥30 kg/m^2^.

^a^ Data are shown as median and 25 ^th^ and 75 ^th^ percentile (p25-p75).

^b^ Data are shown in frequencies and percentages. Chi-square test.

^c^ Data are shown as median (minimum and maximum).

^*^Mann Whitney *U* Test. The level of significance was set at *p* < 0.05.

**FIGURE 1:**
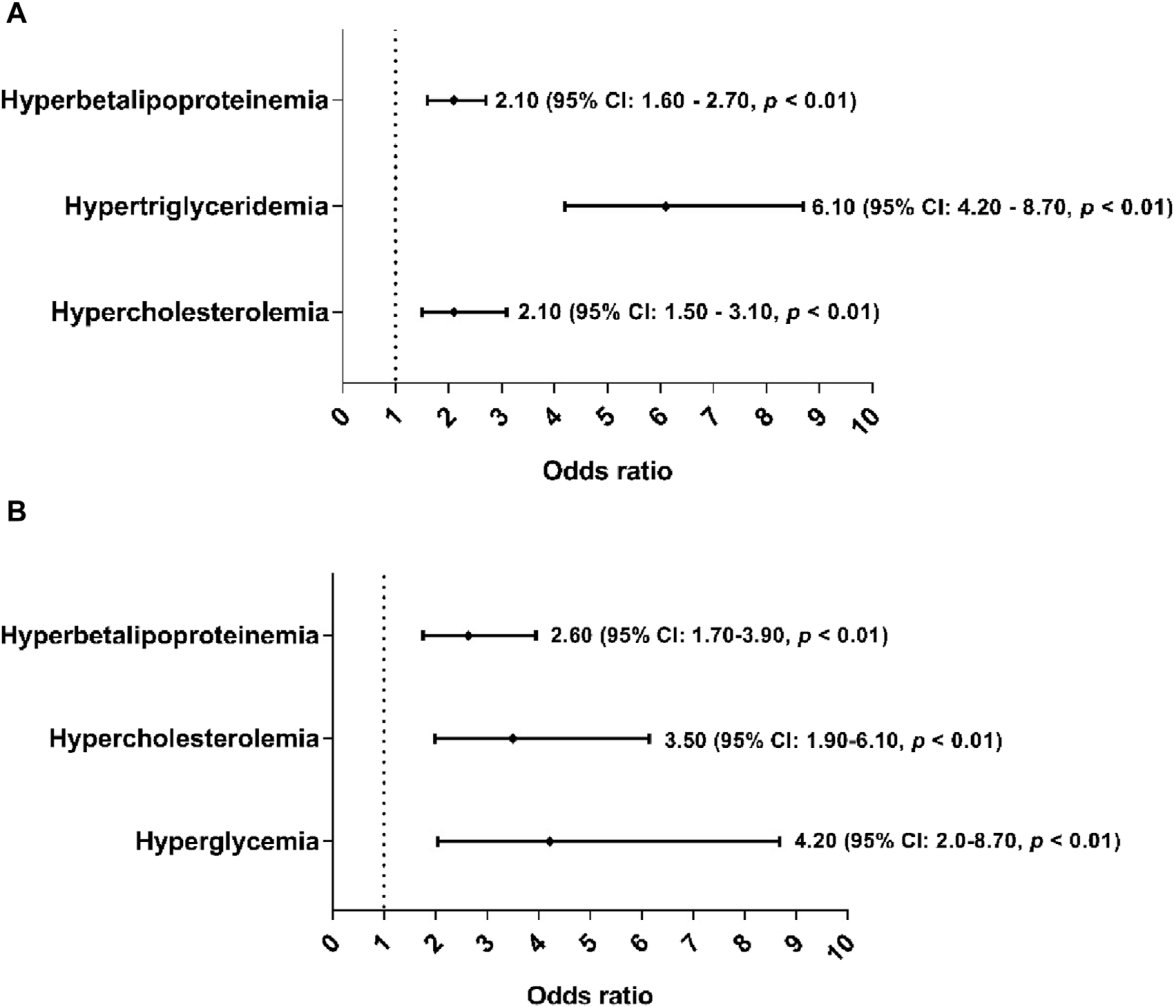
Association of hyperbetalipoproteinemia, hypercholesterolemia, hypertriglyceridemia and hyperglycemia as cardiometabolic risk factors of overweight and obesity. The cutoff criteria were based on to Mexican and National Cholesterol Education Program Adult (ATP III) guidelines. The overweight and obesity group were based on of the cutoff criteria according to the World Health Organization. **(A)** Association of cardiometabolic risk factors in subjects with overweight. **(B)** Association of cardiometabolic risk factors in subjects with obesity.

Furthermore, overweight and obese subjects were associated with hypertriglyceridemia, hypercholesterolemia, and hyperglycemia, where overweight subjects were up to six times more likely to have hypertriglyceridemia (OR: 6.10%, 95% CI: 4.20–8.70, *p* < 0.01) (Figure 1A). While obese subjects were four times more likely to have hyperglycemia (Figure 1B) in both sexes (OR: 4.20%, 95% CI: 2.0–8.7, *p* < 0.01).

**FIGURE 2:**
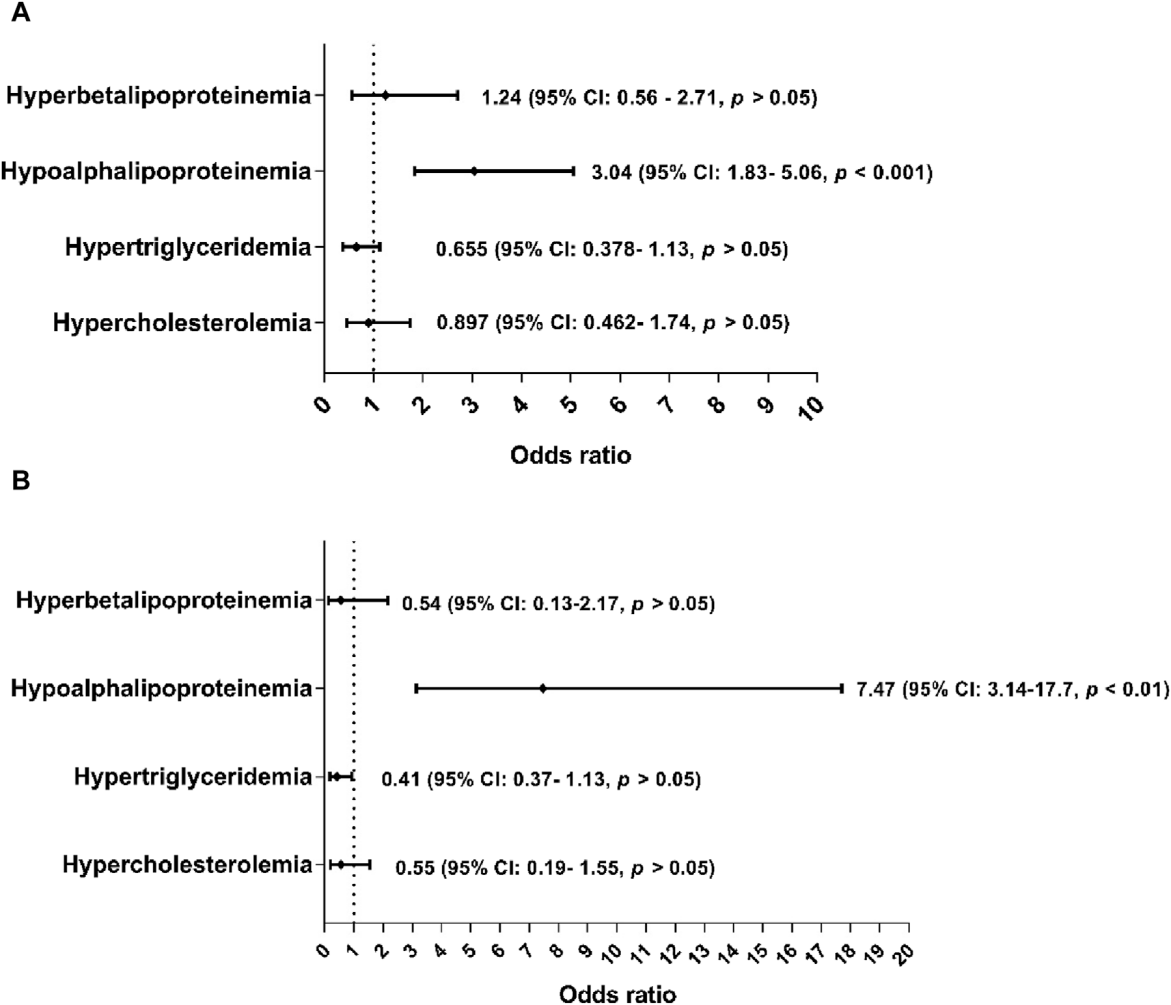
Association of hyperbetalipoproteinemia, hypercholesterolemia, and hypertriglyceridemia as cardiometabolic risk factors of overweight and obesity according to sex. Odds ratio for the sex condition where male sex was considered as a cardiometabolic risk factor. The cutoff criteria were based on to Mexican and National Cholesterol Education Program Adult (ATP III) guidelines where hypoalphalipoproteinemia: ≤40 mg/dL in male sex and ≤50 mg/dL in female sex. The overweight and obesity group were based on of the cutoff criteria according to the World Health Organization. **(A)**: Association of cardiometabolic risk factors in subjects with overweight. **(B)**: Association of cardiometabolic risk factors in subjects with obesity.

In the evaluation by sex, it was observed that the male sex was a risk group for suffering from cardiometabolic disease, unlike the female sex. It was also found that the overweight and obese male sex was three times more likely to present hypoalphalipoproteinemia (OR: 3.04; 95% CI: 1.83–5.06; *p* < 0.001) than overweight females (OR: 7.47; 95% CI: 3.13–17.7; *p* < 0.001) (Figures 2A,B).

### Determination of allelic and phenotypic frequencies

The most prevalent genotypic frequency in this population for rs7895833 (A>G) and rs1467568 (A>G) was the heterozygous group (AG), and the most frequent allele was the A allele for both SNVs (Table 2). The frequencies found in the population of San Luis Potosí, Mexico were compared with the frequencies in Mexican American populations of the Ensembl project database, the results were similar, and no significant differences were found. For example, for the SNV rs7895833, the frequencies of alleles A and G in our population were 61% and 39%, respectively, while for the Mexican-American population, the allelic frequencies of A and G were 61.7% and 38.3%, respectively.

**TABLE 2:** Genotype frequencies of the study population.

*SIRT 1*		n (%)
rs7895833 (A > G)		
Genotypes		
	**AA**	145 (35.8)
	**AG**	204 (50.4)
	**GG**	55 (13.8)
**Alleles**		
	**A**	494 (61.0)
	**G**	315 (39.0)
rs1467568 (A > G)		
**Genotypes**		
	**AA**	149 (36.8)
	**AG**	194 (47.9)
	**GG**	62 (15.3)
**Alleles**		
	**A**	492 (60.7)
	**G**	318 (39.3)

*SIRT, 1:* Sirtuin 1.

Data are shown in frequencies and percentages.

With respect to the SNV rs1467568, the allele frequencies of A and G in our population were 60.7% and 39.3%, respectively, and the frequencies in Mexican Americans were 61.7% (A) and 38.3% (G).

According to the Hardy-Weinberg analysis, both *SIRT1* SNVs were found in equilibrium (*p* > 0.05) for all the groups analyzed (Table 3). Furthermore, LD analysis showed a low LD with a D′ value of 0.58, indicating that both of the above *SIRT1* SNVs had a 58% probability of being inherited together from generation to generation.

**TABLE 3:** Genotypic and allelic frequencies of the study participants classified by body mass index.

	Study population *n* = 809		Normal weight *n* = 603		Overweight group *n* = 152		Obesity group *n* = 54	
	rs7895833	rs1467568	rs7895833	rs1467568	rs7895833	rs1467568	rs7895833	rs1467568
Genotypic frequencies (A > G)								
**AA**	0.358	0.368	0.358	0.350	0.433	0.424	0.373	0.269
**AG**	0.504	0.479	0.481	0.483	0.450	0.454	0.475	0.499
**GG**	0.138	0.153	0.161	0.167	0.117	0.122	0.151	0.232
Allelic frequencies (A > G)								
**A**	0.610	0.607	0.598	0.591	0.658	0.651	0.611	0.519
**G**	0.390	0.393	0.402	0.409	0.342	0.349	0.389	0.409
Hardy Weinberg Equilibrium^a^	*p* = 0.238	*p* = 0.929	*p* = 0.610	*p* = 0.987	*p* = 0.334	*p* = 0.701	*p* = 0.380	*p* = 0.841

Normal weight: ≥18.5 kg/m^2^; Overweight: body mass index ≥25–29 kg/m^2^; Obesity: body mass index ≥30 kg/m^2^.

^a^ Chi-square test.

### Differential association of alleles and genotypes of SNVs with biochemical factors

When evaluating the frequencies of the SNVs rs7895833 and rs1467568 *SIRT1* haplotypes, we found that the most frequent haplotype was A/G in the OG/OBG group while the highest frequency for the haplogenotype was AG/AA in the same group. When performing the association analysis, we found that no haplotype or haplogenotype was significantly associated with overweight and obesity, considering the A/A haplotype and AA/AA haplogenotype as the reference group for not having the risk allele (G) of the SNVs (Table 4) and when analyzed using the dominant genetic model there were no significant associations either (Supplementary Table S1).

**TABLE 4:** Haplotype association of rs7895833 and rs1467568 in the *SIRT 1* gene con overweight/obese and normal weight.

SIRT1 haplotypes	OG/OBG n (%)	NW n (%)	OR (95% CI)^a^	p*
AA	61 (29.7)	165 (28.3)	1	
AG	72 (34.9)	188 (32.2)	1.07 (0.76–1.50)	0.709
GA	66 (32)	189 (32.3)	0.93 (0.66–1.31)	0.661

SIRT1 haplogenotypes	OG/OBG n (%)	NW n (%)	OR (95% CI)^a^	p*
AA/AA	7 (1.8)	20 (5.1)	1	
AA/GG	14 (3.5)	35 (8.9)	1.14 (0.40–3.30)	0.805
GG/AA	7 (1.8)	19 (4.8)	1.05 (0.31–3.57)	0.934
GG/GG	0 (0)	4 (1)	0.71 (0.07–7.53)	0.779
AG/AA	26 (6.6)	65 (16.4)	1.14 (0.43–3.03)	0.788
AG/GG	2 (0.5)	3 (0.70)	1.90 (0.36–13.9)	0.520
AA/AG	19 (4.8)	48 (12.1)	1.13 (0.41–3.11)	0.811
GG/AG	3 (0.7)	19 (4.8)	0.45 (0.10–2)	0.288
AG/AG	25 (6.3)	79 (20.2)	0.90 (0.34.2.39)	0.839

OG: overweigh group; OBG: obesity group; NW: normal weight. For the purposes of this study haplotypes whose frequency was less than 1% were removed.

^a^ Data are shown as odd ratio (95% Confidence Interval).

^*^Chi-square test. The level of significance was set at *p* < 0.05.

When evaluating the association with anthropometric, clinical, and biochemical parameters, we found that a trend for non-risk effect was obtained between rs1467568 *SIRT1* SNV and LDL cholesterol levels (OR = 0.66; 95% CI = 0.43–1; *p* = 0.05) (Supplementary Table S2). The female sex carrying the G allele of rs1467568 had lower levels of cholesterol, triglycerides, and LDL cholesterol than female homozygous for the A allele (Table 5).

**TABLE 5:** Anthropometric, clinical and biochemical characteristics stratified according by dominant model according to *SIRT 1* rs1467568 SNV.

	Study population			Female			Male		
Parameter	AA (n = 148)	AG + GG (*n* = 257)	*p* *	AA (*n* = 59)	AG + GG (*n* = 101)	*p* *	AA (*n* = 86)	AG + GG (*n* = 159)	*p* *
Anthropometric (kg/m^2^)									
Body mass index	22.7 (20.7–25.4)	22.7 (20.8–24.9)	0.490	22.7 (20.4–25.2)	22.5 (20.7–24.6)	0.927	22.9 (21–25)	22.7 (20.8–25.2)	0.621
Clinical (mmHg)									
SBP	100 (100–110)	110 (100–110)	0.039	100 (90–110)	100 (100–110)	0.090	110 (100–112)	110 (100–110)	0.779
DBP	70 (60–80)	70 (60–80)	0.176	60 (60–70)	70 (60–70)	0.256	70 (60–80)	70 (70–80)	0.727
Biochemical (mg/dL)									
Glucose	86 (81–92)	87 (81.5–91)	0.995	85.5 (80–90)	85 (80–90)	0.823	89 (82–93)	87 (83–93)	0.656
Cholesterol	165 (146–189)	162 (146–178)	0.138	175 (152–189)	163 (148–176)	0.030	160 (145–177)	159 (145–182)	0.664
Triglycerides	86 (66–109)	84 (63–112)	0.543	90 (74.5–105.25)	77 (58–102)	0.026	82 (62.7–111)	88 (65–126)	0.254
HDL Cholesterol	54.8 (47.8–62.2)	52.7 (45.5–62.2)	-	56.1 (49.5–62.8)	56.9 (48.9–65.4)	0.820	52.5 (45.5–61)	51.9 (45.4–60)	0.477
LDL Cholesterol	93.3 (79.1–113)	89.1 (75–104)	0.066	97.1 (81.3–113)	87.1 (76.8–103)	0.013	90.5 (74–103)	89.5 (76–109)	0.640

SBP: systolic blood pressure; DBP: diastolic blood pressure.

Data are shown as median and 25th and 75th percentile (p25-p75).

^*^Mann Whitney *U* Test. The level of significance was set at *p* < 0.05.

SNV rs7895833 of *SIRT1* was not associated with anthropometric, clinical or biochemical parameters. However, we observed that in the overweight group the study population as well as the male sex carrying the G allele for SNV rs7895833 had slightly lower BMI levels compared to the group homozygous for the A allele (*p* = 0.009) (Table 6). While for the group with obesity, an increase in total cholesterol and LDL cholesterol levels was observed in the group carrying the G allele, however it was not significant when classified by sex (Table 6).

**TABLE 6:** Anthropometric, clinical and biochemical characteristics stratified by sex according to SNV rs7895833 genotypes of *SIRT 1* in overweight and obese subjects.

Parameter	**Obesity group**								
	**Study population**			**Female**			**Male**		
	**AA (n = 9)**	**AG + GG (n = 18)**	p*	**AA (n = 4)**	**AG + GG (n = 4)**	p*	**AA (n = 5)**	**AG + GG (n = 14)**	p*
Anthropometric (Kg/m^2^)									
Body mass index	27.2 (26.0–28.8)	26.2 (25.6–27.5)	0.009	27.2 (26–28.8)	26.4 (25.9–28.5)	0.404	27 (26.8–28.8)	26 (25.5–27.2)	0.009
Clinical (mmHg)									
SBP	110 (100–120)	110 (100–110)	0.094	110 (100–110)	110 (100–110)	0.711	110 (100–120)	110 (100–110)	0.077
DBP	80 (70–80)	70 (65–80)	0.272	70 (60–80)	70 (60–80)	0.677	80 (70–80)	70 (70–80)	0.302
Biochemical (mg/dL)									
Glucose	90 (83–92)	88 (82–92)	0.673	86 (80–91)	89 (82–92)	0.547	90 (84–92)	87 (81.7–90.7)	0.268
Cholesterol	168 (158–189)	177 (160–191)	0.254	174 (163–212)	185 (173–191)	0.611	164 (154–177)	167 (151–191)	0.488
Triglycerides	112 (84–147)	105 (83–141)	0.783	113 (95–158)	101 (89.5–140)	0.306	104 (76–127	108 (68.2–150)	0.770
Cholesterol HDL	49.2 (41–59.1)	50.1 (43.5–56.5)	-	46.4 (40–50.6)	53.6 (47–61.2)	0.059	51.3 (42–59)	47.5 (39.6–55.8)	0.169
Cholesterol LDL	96.4 (80.4–107)	102 (88–119)	0.173	106 (83.7–50.6)	102 (93.8–123)	0.853	93.5 (75–102)	102 (78.9–116)	0.135

Parameter	**Obesity group**								
	**Study population**			**Female**			**Male**		
	**AA (n = 9)**	**AG + GG (n = 18)**	p*	**AA (n = 4)**	**AG + GG (n = 4)**	p*	**AA (n = 5)**	**AG + GG (n = 14)**	p*
Anthropometric (Kg/m^2^)									
Body mass index	31.3 (30.3–32.7)	33.2 (31–37.6)	0.111	30 (30.2–31.6)	31 (30.4–35.8)	0.343	32.3 (30.8–35)	34.3 (31.2–38.3)	0.391
Clinical (mmHg)									
SBP	110 (105–125)	120 (110–130)	0.233	105 (92–110)	115 (95–120)	0.343	120 (115–140)	120 (117–132)	1.000
DBP	80 (65–80)	80 (80–87)	0.111	70 (62.5–77.5)	75 (62.5–80)	0.686	80 (70–80)	80 (77.5–90)	0.343
Biochemical (mg/dL)									
Glucose	85 (81.5–98.5)	93.5 (86.7–101)	0.157	84.5 (78–98)	89 (86.5–93)	0.343	86 (81.5–98.5)	96 (86–101)	0.257
Cholesterol	159 (149–184.5)	197 (166–221)	0.045	178 (162–184)	195 (159–215)	0.343	153 (144–188)	200 (166–234)	0.056
Triglycerides	115 (86–220)	160 (95.5–210)	0.681	151 (99–204)	97.5 (80.7–200)	0.686	115 (59.5–243)	163 (119–229)	0.444
Cholesterol HDL	47.1 (43.2–51.55)	48.5 (43–53.9)	-	46.5 (42–57)	56.2 (43.5–74.9)	0.486	47 (42.7–51.5)	46.9 (43–52.7)	0.754
Cholesterol LDL	89.1 (76–104.55)	115 (93.7–134)	0.021	100 (82.7–105)	104 (95.6–119)	0.486	87.2 (72–103)	119 (91.1–142)	0.056

SBP: systolic blood pressure; DBP: diastolic blood pressure.

Data are shown as median and 25th and 75th percentile (p25-p75).

^*^Mann Whitney *U* Test. The level of significance was set at *p* < 0.05.

## Discussion

SNVs could influence the severity of diseases such as overweight and obesity. Specifically, some genetic variants in the *SIRT1* gene have been associated with elevated BMI levels and alterations in biochemical and clinical parameters. In the present work, two *SIRT1* SNV, the rs1467568 and rs7895833, were analyzed in a young adult population from the northeastern region of Mexico. We found that these *SIRT1* SNVs were not associated with clinical, biochemical, and anthropometric parameters.

In this study population, the A allele was found to be the most frequent allele for both *SIRT1* SNV. The male sex carrying the SNV rs7895833 (G allele) presented a lower BMI than subjects without the this *SIRT1* SNV and subjects carrying the *SIRT1* SNV rs1467568 (G allele) showed a non-risk effect with a 34% probability of not presenting hyperbetalipoproteinemia, where women carrying the rs1467568 had lower levels of total cholesterol, triglycerides, and LDL cholesterol than women without this SNV.

The allelic and genotypic frequencies in the population of San Luis Potosí were like the frequencies reported in the Mexican American population from the Ensembl project database.

However, differences in the allelic frequencies were observed when comparing our results with studies in other populations; in addition, to the fact that in the Asian population, the most frequent allele is the G allele (Shimoyama et al., 2012; De Oliveira Meneguette et al., 2016). These differences may be due to the type of sampling, sample size, and genetic background of each population studied.

\Regarding the SNVs rs1467568 and rs7895833, it has been described that Japanese female carrying the G allele of rs1467568 or the A allele of rs7895833 had a higher BMI than female without these SNVs (Higashibata et al., 2016), and Casarotto et al. found that the G allele of the SNV rs7895833 was associated with an increased risk of dyslipidemia in a Brazilian geriatric population (Casarotto et al., 2019). In our study, obese subjects carrying the G allele of SNV rs7895833 presented higher lipid levels when assessed in both sex. Notably, overweight subjects carrying the *SIRT1* SNV G allele rs7895833 and subjects carrying the *SIRT1* SNV G allele rs1467568 presented less lipid and clinical alterations being similar to an investigation performed in Indian and South African subjects carrying the G allele of rs1467568 and rs7895833, where no associations with clinical or biochemical parameters were found (Ramkaran et al., 2016).

In Dutch subjects carrying the G allele of rs7895833 and the A allele of rs1467568, the risk of obesity was 18% lower, and those SNVs were associated with a lower BMI (Zillikens et al., 2009). These results suggest that both SNVs could have a non-risk effect for cardiometabolic diseases, although ethnicity, BMI, age and sex could affect that relationship.

Therefore, although it is true that the functional effect that both SNVs have on the associated protein is still being investigated, the data obtained in our study indicate that the *SIRT1* genetic variants could have a possible non-risk effect for dyslipidemia. A possible explanation would be that the *SIRT1* protein promotes the reverse transport of cholesterol by acting on the LXRα20 receptors (which have a repressive role in processes such as thermogenesis in brown adipose tissue or cholesterol homeostasis) (Wang et al., 2008). Another possible explanation could be due to the ability of SIRT1 to regulate other anti-aging genes such as Klotho, p66Shc, and the Forkhead box proteins (FOXO1/FOXO3a), which in turn is closely related to development of obesity and other metabolic diseases and could also depend on the SNVs of the *SIRT 1* gene present in the population (Martins, 2016; Martins, 2017). The SIRT1 protein exerts a deacetylation effect on those nuclear receptors in a lysine residue in position 432, resulting in a ubiquitination process and subsequent degradation; this leaves the DNA free to encode the genes for the synthesis of the ABCA1 receptor, which is a transporter that mediates the first steps of HDL cholesterol synthesis (Li et al., 2007). Although the real effect exerted by both SNVs on the *SIRT1* has not been fully identified, the results obtained in this study allow us to suppose that they lead to greater activation of this protein. Also, due to limited information on the functionality of these two variants, it is difficult to conclude whether individuals carrying both uncommon alleles are associated with low or high SIRT1 activity. However, it could be speculated that these SNVs might induce higher SIRT1 activity (Zillikens et al., 2009).

Regarding the differences found in the cardiometabolic variables, the male sex presented a higher cardiometabolic risk than the female sex. This could also be explained by the protective role that female hormones play on atherosclerosis. Specifically, it has been described that estrogens modulate the atherosclerotic protective action through the signaling of the estrogen receptor alpha/SREBP-1, which is a transcription factor of genes related to lipid synthesis **(** Xie et al., 2022 **)** and the potential of SREBP-1 to reduce fatty acid synthesis and enhance insulin secretion has been described in mice (Ruiz et al., 2014), Finally, current Mexican populations are genetically heterogenous as a consequence of migrations and cultural and lifestyle transitions, which contribute to the allele selection and diversity observed in Native American populations (Barquera et al., 2020). For example, one hypothesis mentions that some genes related to metabolic diseases could be the target of natural selection in humans (Rees et al., 2020), and the allelic frequencies of genetic variants such as SNVs could have been affected by the interbreeding derived from the European colonization of the American continent (Lindo et al., 2016).

In fact, García et al. performed a genome-wide scan to detect the signatures of selection in the genomes of Mexican individuals in five regions of Mexico (North, Northwest, Central, South and Southeast), finding that the selected genes were mainly related to metabolic and immunological phenotypes, and the set of these genes under selection were different in each region, highlighting the interpopulation genetic variability (Barquera et al., 2020). This could partly explain our results in the association analysis as well as the differences found in allelic and haplotypic frequencies with respect to other populations.

This study has limitations. For example, haplotype analysis was performed using a static inference method and may not fully represent the link between these genetic variants in germ cells. Therefore, further studies are needed in family groups where there is a clear distribution of genetic variants to demonstrate their LD in the Mexican population. Moreover, subsequent Mendelian randomization studies will be necessary to examine how certain environmental factors modified the outcomes observed in this genetic study to elucidate the interaction of the *SIRT1* variants with the participants’ environmental factors.

Another limitation is that we did not consider other variables such as the physical activity of the participants for our analysis and due to the descriptive nature of the study; therefore, we were not able to establish a search for confounding factors that could influence the results described. However, this study also has strengths, such as the sufficient sample size for statistical power and that it is one of the few studies that explores these two genetic variants in the Mexican population.

As a perspective of this study, it is contemplated to measure the plasma levels of the SIRT1 protein, especially in patients with the GG genotype for the genetic variants of the rs7895833 and rs1467568 *SIRT1*, and to assess whether SIRT1 could be used as a protein marker of diagnosis in various metabolic diseases in the Mexican population. In order to understand the role that different *SIRT1* genetic variants may have in the development and progression of obesity, and its metabolic comorbidities, could be used in conjunction with measuring SIRT1 protein levels as a risk marker and/or diagnosis of these pathologies (Martins, 2018).

In conclusion, carrying the GG genotype of the *SIRT1* rs7895833 genetic variants in overweight subjects and rs1467568 in women could confer a possible non-risk effect against dyslipidemia.

## Data Availability

The data presented in the study are deposited in the figshare repository: http://doi.org/10.6084/m9.figshare.25332040.
