# Supplementary material for: Analysis of SIRT1 genetic variants in young Mexican individuals: relationships with overweight and obesity
Source: Front Genet. 2024 Apr 4;15:1278201. doi: 10.3389/fgene.2024.1278201 (PMC11027998; doi:10.3389/fgene.2024.1278201)
Supplement: Supplementary file 1 [file Table1.DOCX]

Supplementary Material

**Analysis of *SIRT1* genetic variants in young Mexican individuals: Relationships with overweight and obesity**

**Salazar-García S ^1a^., Ibáñez-Salazar A^2a^., Lares-Villaseñor E^1^., Gaytán-Pacheco N^2^., Uresti-Rivera Uresti E ^3^., Portales-Pérez DP^5^., De la Cruz-Mosso U^4^., Vargas-Morales JM^1^.**

*1. Laboratorio de Análisis Clínicos. Facultad de Ciencias Químicas, Universidad Autónoma de San Luis Potosí. México.*

*2. Unidad Académica de Ciencias Químicas, Universidad Autónoma de Zacatecas ¨Francisco García Salinas. México.*

*3. Laboratorio de Biología.* *Facultad de Ciencias Químicas, Universidad Autónoma de San Luis Potosí. México.*

*4. Red de Inmunonutrición y Genómica Nutricional en las Enfermedades Autoinmunes; Instituto de Neurociencias Traslacionales, Departamento de Neurociencias, Centro Universitario de Ciencias de la Salud; Universidad de Guadalajara, México.*

*5. Centro de Investigación en Ciencias de la Salud y Biomedicina, Universidad Autónoma de San Luis Potosí. México.*

^a^These authors contributed equally to the work.

# Supplementary table 1. Association of rs7895833 and rs1467568 risk alleles in the *SIRT1* gene with overweight/obesity and normal weight.

| Single Nucleotide Variants | OG/OBG  n=103 | NW  n=302 | OR (CI 95%) ^a^ | *p^*^* |
| --- | --- | --- | --- | --- |
| rs7895833 | **n (%)** | **n (%)** |  |  |
| Genotype  AA  AG  GG | 40 (9.9)  53 (13)  10 (2.5) | 105 (25.9)  151 (37.3)  46 (11.4) | 1  0.92 (0.57-1.49)  0.57 (0.26-1.24) | 0.738  0.153 |
| Allele  A  G | 133 (16.4)  73 (9) | 361 (44.6)  243 (30) | 1  0.81 (0.59-1.13) | 0.223 |
| Dominant genetic model  AA  AG + GG | 40 (9.9)  63 (15.5) | 105 (25.9)  197 (48.7) | 1  0.84 (0.53-1.33) | 0.457 |
| rs1467568 |  |  |  |  |
| Genotype  AA  AG  GG | 40 (9.9)  47 (11.6)  16 (3.9) | 109 (26.9)  147 (36.3)  46 (11.4) | 1  0.87 (0.53-1.42)  0.95 (0.48-1.86) | 0.581  0.876 |
| Allele  A  G | 127 (15.7)  79 (9.7) | 365 (41.1)  239 (29.5) | 1  0.95 (0.69-1.31) | 0.757 |
| Dominant genetic model  AA  AG+GG | 40 (9.9)  63 (15.5) | 109 (26.9)  193 (47.7) | 1  0.90 (0.57-1.43) | 0.618 |

OG: overweigh group; OBG: obesity group; NW: normal weight.

^a^Data are shown as odd ratio (95% Confidence Interval).

*Chi-square test. The level of significance was set at *p* < 0.05.

**Supplementary** **table 2. Association of rs1467568 risk alleles in the *SIRT1* gene with hyperbetalipoproteinemia**

| Condition | AG + GG  n= 257 | AA  n= 148 | OR^a^ | CI 95% | *p*^*^ |
| --- | --- | --- | --- | --- | --- |
|  | **n (%)** | **n (%)** |  |  |  |
| Without hyperbetalipoproteinemia  With hyperbetalipoproteinemia | 179 (44.2)  78 (19.2) | 89 (21.9)  59 (14.7) | 0.66 | 0.43 – 1.00 | 0.051 |

Hyperbetalipoproteinemia defined as LDL cholesterol levels > 100 mg/dL.

^a^Data are shown as odd ratio (95% Confidence Interval).

*Chi-square test. The level of significance was set at *p* < 0.05.

**
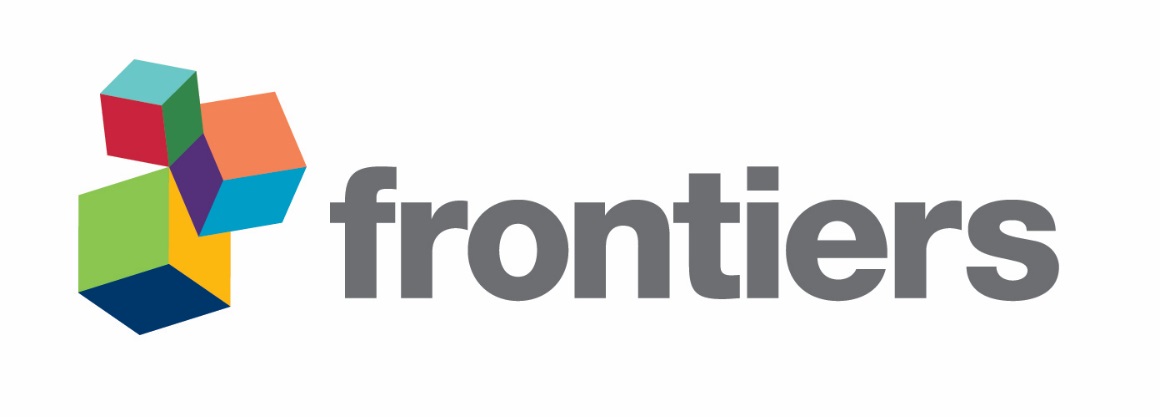
**
